# Infective Endocarditis by *Lactobacillus* Species—A Narrative Review

**DOI:** 10.3390/antibiotics13010053

**Published:** 2024-01-04

**Authors:** Petros Ioannou, Afroditi Ziogou, Ilias Giannakodimos, Alexios Giannakodimos, Stella Baliou, George Samonis

**Affiliations:** 1 School of Medicine, University of Crete, 71003 Heraklion, Greece; 2 School of Medicine, National and Kapodistrian University of Athens, 11527 Athens, Greeceiliasgiannakodimos@gmail.com (I.G.);; 3 First Department of Medical Oncology, Metropolitan Hospital of Neon Faliron, 18547 Athens, Greece

**Keywords:** infective endocarditis, *Lactobacillus*, vegetation, bacteremia

## Abstract

Bacteria of the genus *Lactobacillus* are microaerophilic or aerotolerant anaerobic Gram-positive non-spore-forming rods. They are considered essential members of the human gut microbiome; however, recent studies have revealed that these microorganisms are less predominant in the gut microbiome than initially thought. *Lactobacillus* spp. is mainly known for its use as a probiotic in foods and supplements to prevent and treat specific issues such as infectious diseases, irritable bowel syndrome, and diabetes mellitus. However, *Lactobacillus* spp. may occasionally cause infections such as bacteremia or infective endocarditis (IE). The present study aimed to review all cases of IE by *Lactobacillus* spp. and describe the epidemiology, microbiology, clinical characteristics, treatment, and outcomes of this infection by collecting relevant data from studies existing in Pubmed and Scopus until 28 September 2023. A total of 77 studies containing data for 82 patients were included. The median age was 56 years, and 69.6% were male. A prosthetic valve was present in 16% of patients, and 17.3% had previously been on probiotics. The aortic valve was the most commonly involved intracardiac site, followed by the mitral valve. Fever, embolic phenomena, sepsis, and heart failure were the most common clinical presentations. Aminoglycosides and penicillin were the most commonly used antimicrobials for definitive treatment. Surgery was performed in 53.7% of patients. Overall mortality was 17.1%. IE in prosthetic valves and presentation with shock were independently associated with overall mortality.

## 1. Introduction

Infective Endocarditis (IE) is an infection that involves the endocardium or prosthetic material in the heart, like prosthetic heart valves or cardiac implantable electronic devices (CIED, such as defibrillators and pacemakers). IE carries significant morbidity and mortality [1,2]. It is classically caused by aerobic Gram-positive cocci, such as streptococci, staphylococci, and enterococci, that may add up to 75% of the implicated microorganisms in patients with IE [3,4]. However, IE may also occur in the context of anaerobic bacteria, Gram-positive rods, or Gram-negative bacteria. The exact characteristics of IE by these pathogens have not been adequately described due to the rarity of the disease that they cause [5,6,7].

Bacteria of the genus *Lactobacillus* are microaerophilic or aerotolerant anaerobic Gram-positive non-spore-forming rods. They are considered essential members of the human gut microbiome, especially the oral cavity and ileum, and the female genital tract [8,9]; however, recent studies have revealed that these microorganisms are not as predominant in the gut microbiome as was initially thought [10]. *Lactobacillus* spp. is widely known for its use as a probiotic in foods and supplements to prevent and treat specific diseases [11,12]. These bacteria may also exert protective roles in the human microbiota, as shown in the case of the female genital tract, where women with a decrease in or absence of *Lactobacillus* spp. are more prone to developing bacterial vaginosis [13]. For example, there is ongoing research regarding these microorganisms’ benefits in infectious diseases, irritable bowel syndrome, inflammatory bowel disease, rheumatoid arthritis, type one diabetes, multiple sclerosis, obesity, type two diabetes, cancer, and cognitive development and behavior [10,11,12]. However, *Lactobacillus* spp. may occasionally cause infections, more commonly in patients with specific risk factors, such as diabetes, structural heart disease, cancer, total parenteral nutrition, chronic kidney disease, organ transplantation, and current antimicrobial use [14,15,16,17,18]. These infections include bacteremia, septic shock, urinary tract, central nervous system, prosthetic joint, and intra-abdominal infections as well as pneumonia, lung abscess, and IE, among others [19]. IE caused by *Lactobacillus* spp. is a rare infection with most evidence deriving from case reports and literature reviews [20].

The present study aimed to review all cases of IE by *Lactobacillus* spp. and describe the epidemiology, clinical characteristics, microbiology, treatment, and outcomes of this infection.

## 2. Methods

This narrative review is an effort to extract and collect existing data on IE by *Lactobacillus* species in humans. The primary aim of the present study was to provide information on the mortality of these infections. Secondary outcomes were to record data on (a) epidemiology, (b) the exact site of infection, (c) the patients’ clinical characteristics, (d) antimicrobial susceptibility, and (e) their treatment. For this review, PubMed/Medline and Scopus databases were searched for eligible articles reporting “*Lactobacillus* AND endocarditis” until 28 September 2023. This narrative review included original reports on infections, such as case reports and case series that provided information on epidemiology, microbiology, treatment, and outcomes of IE by *Lactobacillus* species in humans. Only articles in the English language were included. Reviews, systematic reviews, retrospective studies, and letters to the editor were excluded. Articles with no access to original data and studies referring to animal reports had to be excluded. Moreover, studies with insufficient data and articles without information on patients’ mortality and epidemiology were also excluded. The remaining articles’ references were also searched to assess potential studies.

Three investigators (I.G., A.G. and A.Z.) extracted information from all the eligible studies for this narrative review using a pre-defined template. The extracted data included study type, year of publication, and country; patient demographic data (age and gender); patients’ relevant medical history (previous cardiac surgery or cardiac valve replacement, time after cardiac valve replacement); infection data and microbiology (infection site, data regarding microorganism identification, presence of complications, presence of embolic phenomena); antimicrobial treatment administered (as definitive treatment); whether surgery was performed, and outcomes (i.e., cure or death). Data on microbiology and the association of mortality with index infection were reported by the original studies’ authors. Overall mortality was noted during admission and the months following patient discharge, as reported by the original studies’ authors. Information about patients’ previous history, oral hygiene, use of antimicrobials, and probiotics was collected as provided by the studies’ authors. If no such information was provided, the particular characteristic was considered as not being present in the patient’s history. Diagnosis of IE was confirmed by the investigators based on information provided by the authors and the modified Dukes’ criteria if the diagnosis was at least possible (at least one major and one minor criterion or at least three minor criteria) or if pathological data established a diagnosis of IE [21].

Data are presented as numbers (%) for categorical variables and mean (standard deviation) or median (interquartile range, IQR) for continuous variables. Categorical data were analyzed with Fisher’s exact test. Continuous variables were compared using the Mann–Whitney U-test for non-normally distributed variables or the t-test for normally distributed variables. All tests were two-tailed, and a *p*-value equal to or lower than 0.05 was considered significant. A univariate linear regression analysis was conducted to identify factors associated with all-cause mortality of patients with IE. More specifically, a univariate logistic regression was performed to identify any association between gender, age, presence of prosthetic cardiac valve, of poor teeth and oral hygiene or recent dental work, of previous episode of IE, rheumatic heart disease history, presence of IE at the aortic, mitral, pulmonary, tricuspid valve, presence of IE at multiple valves, presentation with fever, sepsis, embolic phenomena, development of heart failure, treatment with particular antimicrobials and performed surgical management with all-cause mortality. All statistics were calculated with GraphPad Prism 6.0 (GraphPad Software, Inc., San Diego, CA, USA). A multivariate logistic regression analysis evaluated the effect of factors previously identified in the univariate analysis model associated with all-cause mortality with a *p* < 0.05. Multivariate analysis was performed using the SPSS version 23.0 (IBM Corp., Armonk, NY, USA).

## 3. Results

### 3.1. Included Studies’ Characteristics

**Figure 1:**
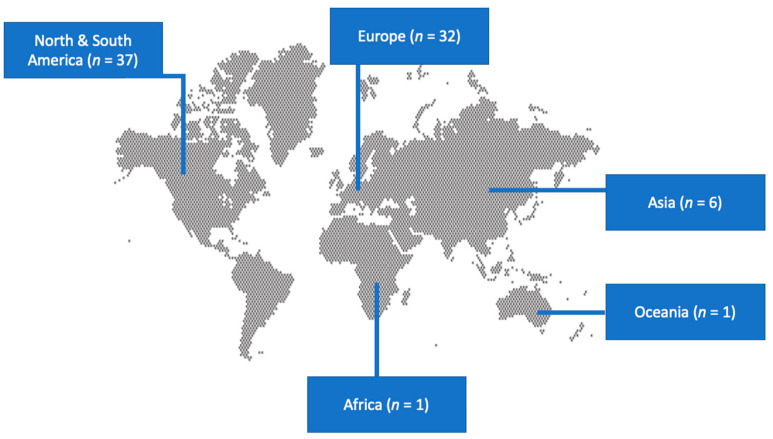
Geographical distribution of studies on infective endocarditis by *Lactobacillus* species worldwide.

**Figure 2:**
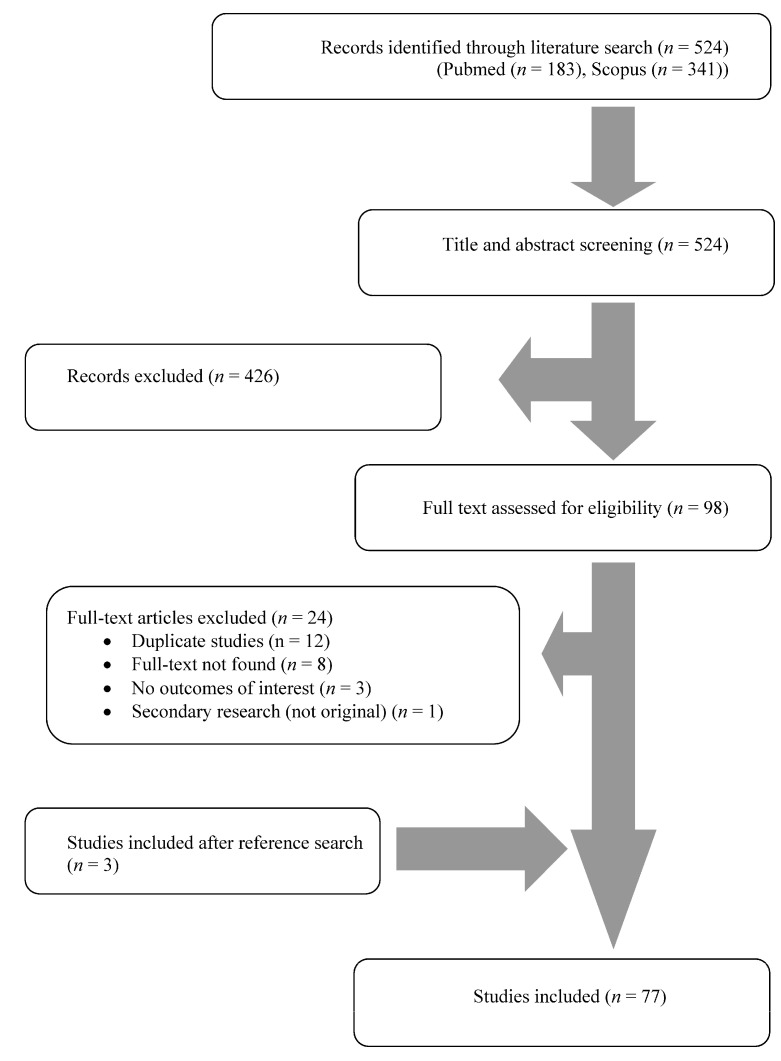
Flow diagram of study inclusion.

A total of 485 articles from PubMed and Scopus were screened. Finally, 77 met the present study’s inclusion criteria [20,22,23,24,25,26,27,28,29,30,31,32,33,34,35,36,37,38,39,40,41,42,43,44,45,46,47,48,49,50,51,52,53,54,55,56,57,58,59,60,61,62,63,64,65,66,67,68,69,70,71,72,73,74,75,76,77,78,79,80,81,82,83,84,85,86,87,88,89,90,91,92,93,94,95,96,97]. The 77 studies included in the current narrative review involved 82 patients in total. Among these studies, 37 were conducted in North and South America, 32 in Europe, 6 in Asia, 1 in Africa, and 1 in Oceania. There were 70 case reports and seven case series. Figure 1 shows the geographical distribution of *Lactobacillus* species worldwide and Figure 2 shows the flow diagram of study inclusion.

### 3.2. Epidemiology of IE by Lactobacillus Species

The age of patients with IE by *Lactobacillus* species ranged from 2 to 85 years; the median age was 56 years, and 69.6% (55 out of 79 patients with available data) were male. Regarding predisposing factors, 41.5% (34/82) had poor teeth hygiene or recent dental work, 19% (15/79) had received antimicrobials within the last three months, 17.3% (14/81) had received probiotics during the previous three months, 16% (13/81) had a prosthetic cardiac valve, 9.8% (8/82) had a previous episode of IE, 8.5% (7/82) had history of congenital heart disease, 7.3% (6/82) had history of immunosuppression, 6.1% (5/82) had history of intravenous drug use (IVDU). Table 1 shows the patients’ characteristics.

### 3.3. Microbiology and Diagnosis of IE by Lactobacillus Species

IE by *Lactobacillus* species was polymicrobial in 6.1% (five patients), with blood cultures being also positive for *Streptococcus* spp. in 2.4% (two), fungi in 2.4% (two), and *Prevotella* spp. in 1.2% (one). The isolated species from the 82 patients with IE were *L. rhamnosus* in 23.2% (19 patients), *L. casei* in 13.4% (11), *L. jensenii* in 13.4% (11), *L. acidophilus* in 13.4% (11), *L. plantarum* in 9.8% (8), *L. paracasei* in 8.5% (7), *L. zeae* in 2.4% (2), *L. salicinius* in 1.2% (1), *L. garvieae* in 1.2% (1), *L. curvatus* in 1.2% (1), *L. salivarius* in 1.2% (1). The species were not identified in 18.8% (12 patients). Antimicrobial resistance to penicillin was 8.5% (4 out of 47 patients with available data), and to aminoglycosides was 15.2% (5). Antimicrobial resistance to vancomycin was 86.2% (25/29).

The diagnosis was facilitated by transthoracic echocardiography in 45.3% (34/75), transesophageal echocardiography in 38.7% (34/75), autopsy in 6.3% (5/80), valve culture in 18.8% (15/80), and by foreign body culture in 6.3% (2/80) of patients. Blood cultures were positive in 97.6% (80/82).

### 3.4. Clinical Characteristics of IE by Lactobacillus Species

The infection affected the aortic valve in 50.7% (39 out of 77 patients with available data), the mitral in 48.1% (37/77), the tricuspid in 2.6% (2/77), the pulmonary valve in 2.6% (2/77), and a cardiac implanted electronic device in 1.2% (1/81). Multiple valves were infected in 5.2% (4/77).

The most common clinical symptoms included fever in 65% (52/80), sepsis in 27.8% (22/79), embolic phenomena in 34.6% (28/81), heart failure in 23.8% (19/80), and shock in 6.3% (5/79) of patients. Table 2 shows the clinical characteristics of patients with IE as well as the definitive treatment administered.

### 3.5. Treatment and Outcomes of IE by Lactobacillus Species

Definitive treatment of patients is summarized in Table 2 and detailed in Table S1. The median treatment among survivors was six weeks (interquartile range: 6–7 weeks). The most commonly used antimicrobials (as definitive treatment) were aminoglycosides in 67.1% (53 out of 79 patients with available data), penicillin in 59.5% (47), aminopenicillin in 39.2% (31), vancomycin in 24.1% (19), and cephalosporins in 20.3% (16) of cases. Surgical management in combination with antimicrobial treatment was performed in 53.7% (44/82) of cases. Overall mortality was 17.1% (14/82), and mortality directly attributed to the IE episode was 12.2% (10). Overall mortality occurred in a median of 12 days (interquartile range 1–46.5 days) after admission to the hospital. As shown in Figure S1, there was a trend for a relative reduction in mortality of patients with IE by *Lactobacillus* spp. as decades went by, with an overall mortality of 33.3% in the decade of 1970–1979 and an overall mortality of 11.8% in the current decade (2020 and on).

### 3.6. Statistical Analysis of IE by Lactobacillus Species

Table 1 and Table 2 compare patients with IE by *Lactobacillus* species who survived with those who died. Patients who died were more likely to have a prosthetic heart valve and were also more likely to develop shock in a statistically significant way.

Among the different parameters tested in the univariate regression analysis, IE in a prosthetic valve, polymicrobial IE, development of shock, as well as heart failure were positively associated with overall mortality. A multivariate logistic regression model identified IE in a prosthetic valve and the development of shock to be independently associated with overall mortality. Table 3 shows the results of the regression analysis.

## 4. Discussion

The present study described the characteristics of patients who developed IE by *Lactobacillus* species. The intracardiac site most commonly involved was the aortic valve, followed by the mitral valve. The most common clinical presentation included fever, embolic phenomena, sepsis, and the development of heart failure. Aminoglycosides and penicillin were the most commonly used antimicrobials for definitive treatment. Overall mortality was 17.1%.

The median age of patients diagnosed with IE by *Lactobacillus* spp. in the present study was 56 years, which is lower than the age in other cohorts of patients with IE, where the mean age is about 70 years [3,4,98]. A male predominance was noted, as is also the case in IE by other microorganisms [3,98]. A prosthetic valve was present in 16% of patients with IE by *Lactobacillus* spp., a rate that is comparable to that of other studies of IE, which at times may be as high as 50% [3,4,98]. A previous episode of IE was noted in 9.8% of patients in the present study, and the rate of patients with a history of rheumatic fever was 6.1%. Both these rates are similar to those of other studies of patients with IE [4,98]. Intravenous drug use was noted in 6.1% of the present patients, which is comparable to that in other studies of IE, where that rate is between 4% and 9.2% [3,4,98]. Congenital heart disease was noted in 8.5% in the present study, being comparable to the corresponding rate in another study of IE in the general population [4]. Notably, 19% of patients with IE by *Lactobacillus* spp. had a previous exposure to antimicrobials, which is consistent with the literature that shows that previous exposure to antimicrobials is a risk factor for the development of infection by *Lactobacillus* spp. [10]. Moreover, 17.3% of the present patients had a previous exposure to *Lactobacillus* spp. in the form of probiotics. Interestingly, the increase in probiotic and dairy consumption and the numerous cases of *Lactobacillus spp*. infections have led to questions regarding probiotics’ safety. The possible relationship between these agents and infection development remains a challenge that has to be proven, since *Lactobacillus spp*. constitutes part of the normal human flora [76]. Indeed, there is ongoing research on the safety of probiotics since, in some cases, mainly in patients with underlying conditions, infections due to probiotics may occur [19,99]. 

The most commonly infected intracardiac sites were the aortic in 50.7% and the mitral valves in 48.1%, which is similar to other studies of IE, where the aortic valve was the most commonly infected valve, followed by the mitral valve [3,98]. In line with the current literature, *L. casei* and *L. rhamnosus* were the most frequently identified pathogens. Regarding clinical presentation, the most common symptom was fever, occurring in 65% of patients, sepsis was evident only in 27.8%, while 6.3% developed shock. In other studies describing patients with IE, fever was present in 84% of patients [4], and shock was diagnosed in 9% [3]. However, fever is absent in almost 40% of *Lactobacillus* IE cases. Heart failure was diagnosed in 23.8% of the present patients, a rate lower than the corresponding rate in other studies evaluating patients with IE, where rates were within the range of 33% to 52% [3,98]. Embolic phenomena in IE by *Lactobacillus* spp. were diagnosed in 34.6% of the present patients, a rate similar to that of other studies with IE in the general population, where it ranged from 15% to 45% [3,4].

*Lactobacillus* species detection by conventional diagnostic methods remains challenging; early clinical suspicion is crucial for an efficient diagnostic approach. Laboratory findings are non-specific and can include thrombocytopenia, monoclonal gammopathy, or a positive rheumatoid factor [93]. In the majority of cases, the diagnostic algorithm *Lactobacillus* IE begins with standard procedures, including blood cultures, transthoracic echocardiogram (TTE), transesophageal echocardiogram (TEE), and application of the Duke’s criteria [91]. TEE constitutes a valuable tool for prompt imaging of valvular regurgitation or valve vegetations, although active infection versus healing vegetations is not easily differentiated. However, in some cases, TEE might not demonstrate findings of endocarditis and, as a result, should be combined with microbiological studies [82]. Valve culture, valves’ PCR, or valve histology also aid in the diagnosis. However, *Lactobacillus* spp. detection in most laboratories is demanding given the problematic culture growth of the pathogen; only 30–50% of the isolates can be identified by conventional methods [69]. Thus, advanced molecular techniques such as 16S rRNA combined with MALDI- TOF MS are required for accurate identification [71,92].

Regarding treating IE by *Lactobacillus* spp., there are no established susceptibility breakpoints; recommendations for antimicrobial administration rely on case series in the current literature. Antimicrobial therapy administered after susceptibility testing significantly decreases mortality rates [90]. Synergistic intravenous therapy with aminoglycosides and penicillin was the most commonly suggested. This is reasonable, considering that resistance to these two antimicrobials was 15.2% and 8.5%, respectively. Aminoglycosides have been used in the treatment of IE in many pathogens, classically in the context of combination therapy, mostly by Gram-positive pathogens, as well as in the case of Gram-negative pathogens [100,101,102,103]. However, there is currently a trend to reduce aminoglycoside use, since it is associated with high morbidity due to kidney injury, while its benefit in mortality is questionable [104,105,106,107,108]. Notably, in the present review, the statistical analysis among patients who survived and those who died did not reveal any statistically significant differences in terms of antimicrobial treatment in general, and for the use of aminoglycosides in the regimen in particular. Furthermore, the univariate linear regression analysis also did not find any association between aminoglycoside use and overall mortality. Thus, it would be tempting to state that aminoglycosides are not necessary in the treatment of this disease. However, only a randomized controlled trial could determine whether aminoglycosides provide any benefit in the treatment of IE by *Lactobacillus* spp. Moreover, this review includes a small number of patients; thus, it does not have adequate power to draw such solid conclusions.

Interestingly, resistance to vancomycin was 86.2%, which is in line with the literature suggesting that treatment of *Lactobacillus* spp. with vancomycin is considered generally ineffective, with most strains of *Lactobacillus* being inherently resistant to this drug [109]. Moreover, cases of resistance to tetracyclines, ciprofloxacin, or carbapenems have also been reported [78]. A possible mechanism of resistance to these agents is lactic acid production, leading to lower pH levels and decreased effectiveness of antimicrobials [71]. Duration of therapy should be per standard guidelines for IE treatment. In the present review, the median intravenous treatment duration was six weeks [78]. In some cases, surgical intervention, such as valve replacement or abscess drainage, should accompany antimicrobial therapy [90]. The presence of heart failure, large mobile vegetations, abscesses, or multi-drug resistant organisms constitutes strong indications for surgical intervention [93].

In the present review, overall mortality was 17.1%, while in 12.2% of all patients, death was directly attributed to the episode of IE. Overall mortality was comparable to the rates noted in other studies of IE, where it was within the range of 11–40% [3,4,98]. Mortality rates are generally attributed to inadequate treatment, polymicrobial infections, and the bacterial pathogenic potential [84]. Importantly, statistical analysis of the cases in the present study identified IE in a prosthetic valve and presentation with shock to be independently associated with overall mortality. Interestingly, a reduction in overall mortality was noted in the studies published more recently. In general, the management of IE has changed over the years, with surgery being more commonly indicated and performed in cases where conservative treatment is inadequate [110]. However, mortality remains high, at 15–30%, which could be attributed to changes in the epidemiology and the microbiology of the disease [110,111,112]. The relative reduction in the overall mortality in patients with IE by *Lactobacillus* spp. may be linked to a similar epidemiology of the disease over the years, as well as medical improvements in the diagnosis and the medical and surgical management of the disease [113,114].

This study has some limitations. First, it mainly consists of case reports; thus, the evidence may be low, since case reports and case series contain sufficient data the credibility of which mainly depends on the accurate record keeping of each institution. For example, the information on the past medical history oral hygiene of patients, the previous use of probiotics, and the previous use of antibiotics relied on a report by each study’s investigator. Inadequate referral could be associated with underreporting of the particular characteristic, leading to bias in the present study. In addition, the heterogeneity among institutions regarding surgical approaches and record-keeping affects information about outcomes and time-to-event analysis. However, given the rarity of this infection, an effort to conduct a prospective or retrospective study evaluating this condition could hardly enroll an adequate number of patients, even if it included cases over many years and from many centers. Finally, this is a narrative, not a systematic review.

## 5. Conclusions

To conclude, this narrative review describes the epidemiology, microbiology, clinical characteristics, treatment, and outcomes of IE by *Lactobacillus* spp. Penicillin and aminoglycosides were the most commonly used antimicrobials for definitive treatment since antimicrobial resistance was low to these antimicrobials. Prosthetic valve IE and presentation with shock were independently associated with mortality.

## Figures and Tables

**Table 1:** Characteristics of patients with infective endocarditis by *Lactobacillus* species in total and regarding patients’ outcomes.

Characteristic	All Patients (*n* = 82) *	Survived (*n* = 68)	Died (*n* = 14)	*p*-Value
Age, years, median (IQR)	53.7 (20)	53.1 (19.9)	56.6 (21.5)	0.4237
Male gender, *n* (%)	55/79 (69.6)	46/67 (68.7)	9/12 (75)	0.7474
Predisposing factors				
Bad teeth hygiene or recent dental work, *n* (%)	34 (41.5)	31 (45.6)	3 (21.4)	0.1375
Previously on antibiotics, *n* (%)	15/79 (19)	14/65 (21.5)	1 (14.3)	0.2847
Previously on probiotics, *n* (%)	14/81 (17.3)	12/67 (17.9)	2 (14.3)	1
Prosthetic valve, *n* (%)	13/81 (16)	8 (11.8)	5/13 (38.5)	0.0303
Previous IE, *n* (%)	8 (9.8)	7 (10.3)	1 (7.1)	1
Congenital heart disease, *n* (%)	7 (8.5)	6 (8.8)	1 (7.1)	1
Immunosuppression, *n* (%)	6 (7.3)	5 (7.4)	1 (7.1)	1
Rheumatic fever, *n* (%)	5 (6.1)	5 (7.4)	0 (0)	0.5821
IVDU, *n* (%)	5 (6.1)	3 (4.4)	2 (14.3)	0.2001
Post cardiac surgery, *n* (%)	2 (2.4)	1 (1.5)	1 (7.1)	0.3141
Method of diagnosis				
Transthoracic echocardiography, *n* (%)	34/75 (45.3)	32/64 (50)	2/11 (18.2)	0.098
Transesophageal echocardiography, *n* (%)	29/75 (38.7)	25/64 (39.1)	4/11 (36.4)	1
Autopsy, *n* (%)	5/80 (6.3)	0 (0)	5/12 (41.7)	NA
Valve culture, *n* (%)	15/80 (18.8)	13 (19.1)	2/12 (16.7)	1
Valve localization				
Aortic valve, *n* (%)	39/77 (50.7)	32/65 (49.2)	7/12 (58.3)	0.7549
Mitral valve, *n* (%)	37/77 (48.1)	33/65 (50.8)	4/12 (33.3)	0.3517
Tricuspid valve, *n* (%)	2/77 (2.6)	2/65 (3.1)	0 (0)	1
Pulmonary valve, *n* (%)	2/77 (2.6)	2/65 (3.1)	0 (0)	1
Multiple valves, *n* (%)	4/77 (5.2)	4/65 (6.2)	0 (0)	1
CIED, *n* (%)	1/81 (1.2)	1 (1.5)	0 (0)	1

CIED: cardiac implanted electronic device; IE: infective endocarditis; IQR: intraquartile range; IVDU: intravenous drug use; NA: not applicable *: data are among the number of patients mentioned on top unless otherwise described.

**Table 2:** Clinical presentation and definitive treatment of patients with infective endocarditis by *Lactobacillus* species in total and regarding patients’ outcomes.

Characteristic	All Patients (*n* = 82) *	Survived (*n* = 68)	Died (*n* = 14)	*p*-Value
Fever, *n* (%)	14 (66.7)	46 (67.6)	6/12 (50)	0.326
Sepsis, *n* (%)	5 (23.8)	16/67 (23.9)	6/12 (50)	0.0834
Heart failure, *n* (%)	4 (19)	13/67 (19.4)	6 (46.2)	0.0689
Embolic phenomena, *n* (%)	5 (23.8)	22 (32.4)	6 (46.2)	0.3561
Shock, *n* (%)	2 (9.5)	1/67 (1.5)	4/12 (33.3)	0.0015
Immunological phenomena, *n* (%)	9/81 (11.1)	9 (13.2)	0 (0)	0.3418
Paravalvular abscess, *n* (%)	4/81 (4.9)	4 (5.9)	0 (0)	1
Definitive Treatment				
Aminoglycoside, *n* (%)	53/79 (67.1)	45/67 (67.2)	8/12 (66.7)	1
Penicillin, *n* (%)	47/79 (59.5)	41/67 (50)	6/12 (50)	0.5319
Aminopenicillin, *n* (%)	31/79 (39.2)	25/67 (37.3)	6/12 (50)	0.5238
Vancomycin, *n* (%)	19/79 (24.1)	15/67 (22.4)	4/12 (33.3)	0.4685
Cephalosporin, *n* (%)	16/79 (20.3)	13/67 (19.4)	3/12 (25)	0.7005
Clindamycin, *n* (%)	7/79 (8.9)	6/67 (9)	1/12 (8.3)	1
Antipseudomonal penicillin, *n* (%)	6/79 (7.6)	6/67 (9)	0/12 (0)	0.5831
Quinolone, *n* (%)	5/79 (6.3)	4/67 (6)	1/12 (8.3)	0.5715
Carbapenem, *n* (%)	4/79 (5.1)	4/67 (6)	0/12 (0)	1
Macrolide, *n* (%)	4/79 (5.1)	3/67 (4.5)	1/12 (8.3)	0.4899
Daptomycin, *n* (%)	2/79 (2.5)	2/67 (3)	0/12 (0)	1
Tetracycline, *n* (%)	2/79 (2.5)	2/67 (3)	0/12 (0)	1
Co-trimoxazole, *n* (%)	1/79 (1.3)	1/67 (1.5)	0/12 (0)	1
Surgical management, *n* (%)	44 (53.7)	39 (57.4)	5 (35.7)	0.1554

*: data are among the number of patients mentioned on top unless otherwise described.

**Table 3:** Regression analysis of overall mortality in patients with infective endocarditis by *Lactobacillus* species.

Characteristic	Univariate Analysis *p*-Value	Multivariate Analysis *p*-Value	OR (95% CI)
Prosthetic valve	0.016	0.018	7.781 (1.429–42.380)
Polymicrobial IE	0.0081	0.111	6.112 (0.660–56.599)
Shock	<0.0001	0.011	28.238 (2.162–368.768)
Heart failure	0.0385	0.167	3.316 (0.606–18.149)

CI: confidence interval; IE: infective endocarditis; OR: odds ratio.

## Data Availability

The data presented in this study are available on request from the corresponding author.

## Supplementary Materials

The following supporting information can be downloaded at: https://www.mdpi.com/article/10.3390/antibiotics13010053/s1, Table S1: Characteristics of the included studies. Figure S1: Overall mortality of patients with infective endocarditis by *Lactobacillus* species over time.
