# Supplementary material for: Infective Endocarditis by Lactobacillus Species—A Narrative Review
Source: Antibiotics (Basel). 2024 Jan 4;13(1):53. doi: 10.3390/antibiotics13010053 (PMC10812763; doi:10.3390/antibiotics13010053)
Supplement: Supplementary file 1 [file antibiotics-13-00053-s001.zip › antibiotics-2806293-supplementary.pdf]

antibiotics

MDPI

Supplementary Materials

Infective Endocarditis by *Lactobacillus* Species—A Narrative Review

Petros Ioannou [1,*], Afroditi Ziogou [2], Ilias Giannakodimos [2,†], Alexios Giannakodimos [2,†], Stella Baliou [1] and George Samonis [1,3,*]

[1] School of Medicine, University of Crete, 71003 Heraklion, Greece
[2] School of Medicine, National and Kapodistrian University of Athens, 11527 Athens, Greece; iliasgiannakodimos@gmail.com (I.G.)
[3] First Department of Medical Oncology, Metropolitan Hospital of Neon Faliron, 18547 Athens, Greece
* Correspondence: p.ioannou@uoc.gr (P.I.); samonis@med.uoc.gr (G.S.)
† These authors contributed equally to this work.

Table S1. Characteristics of the included studies.

Study	Number of patients	Age (years)	Gender	Site of infection *n* (%)	Microbiology of infection *n* (%)	Treatment administered *n* (%)	Infection outcomes *n* (%)
Axelrod et al., 1973 [22]	1	44	Female	NR 1 (100)	*L. plantarum* 1 (100)	Penicillin 1 (100)<br>Aminopenicillin 1 (100)<br>Tetracycline 1 (100)	Clinical cure[a] 1 (100)<br>Deaths overall 0 (0)
Tenenbaum et al., 1975 [23]	1	63	Male	NR 1 (100)	*L. casei* 1 (100)	Penicillin 1 (100)<br>Cephalosporin 1 (100)<br>Aminoglycoside 1 (100)	Clinical cure 1 (100)<br>Deaths overall 0 (0)
Rubenfeld et al., 1977 [24]	1	40	Male	MV 1 (100)	*Lactobacillus* spp. 1 (100)	Penicillin 1 (100)	Clinical cure 1 (100)<br>Deaths overall 0 (0)
Bayer et al., 1978 [25]	3	43, 48, 52	1 female<br>2 male	AoV 3 (100)	*L. plantarum* 2 (66.7)<br>*L. casei* 1 (33.3)	Penicillin 2 (66.7)<br>Aminoglycoside 1 (33.3)<br>No antibiotics 1 (33.3)<br>Surgical management 2 (66.7)	Clinical cure 1 (33.3)<br>Deaths overall 2 (66.7)<br>Deaths due to infection 1 (33.3)
Jawetz et al., 1980 [26]	1	7	Female	TrV 1 (100)	*L. rhamnosus* 1 (100)	Penicillin 1 (100)<br>Aminoglycoside 1 (100)	Clinical cure 1 (100)<br>Deaths overall 0 (0)
Shinar et al., 1984 [27]	1	30	Male	MV 1 (100)	*L. plantarum* 1 (100)	Penicillin 1 (100)<br>Macrolide 1 (100)<br>Rifampicin 1 (100)<br>Aminoglycoside 1 (100)	Clinical cure 1 (100)<br>Deaths overall 0 (0)

						Surgical management 1 (100)	
Sussman et al., 1986 [28]	1	27	Male	AoV 1 (100)	*L. casei* 1 (100)	Penicillin 1 (100) Rifampicin 1 (100) Aminoglycoside 1 (100) Surgical management 1 (100)	Clinical cure 1 (100) Deaths overall 0 (0)
Davies et al., 1986 [29]	2	40, 55	Male	AoV 2 (100) MV 1 (50)	*L. plantarum* 1 (50) *L. casei* 1 (50)	Penicillin 1 (50) Aminopenicillin 1 (50) Aminoglycoside 2 (100)	Clinical cure 1 (50) Deaths overall 1 (50) Deaths due to IE 0 (0)
Fisher et al., 1988 [30]	1	42	Male	AoV 1 (100)	*L. salivarius* 1 (100) *L. salicinius* 1 (100)	Penicillin 1 (100) Rifampicin 1 (100)	Clinical cure 1 (100) Deaths overall 0 (0)
Struve et al., 1988 [31]	1	69	Male	MV 1 (100)	*L. plantarum* 1 (100)	Penicillin 1 (100) Aminoglycoside 1 (100)	Clinical cure 1 (100) Deaths overall 0 (0)
Naude et al., 1988 [32]	1	66	Male	MV 1 (100)	*L. rhamnosus* 1 (100)	Penicillin 1 (100) Aminopenicillin 1 (100) Aminoglycoside 1 (100) Surgical management 1 (100)	Clinical cure 0 (0) Deaths overall 1 (100) Deaths due to IE 1 (100)
Thangkhiew et al., 1988 [33]	1	66	Male	MV 1 (100)	*L. plantarum* 1 (100)	Aminopenicillin 1 (100)	Clinical cure 0 (0) Deaths overall 1 (100) Deaths due to infection 1 (100)
Stulz et al., 1989 [34]	1	72	Male	MV 1 (100)	*Lactobacillus* spp. 1 (100)	Penicillin 1 (100) Surgical management 1 (100)	Clinical cure 1 (100) Deaths overall 0 (0)
Atkins et al., 1990 [35]	1	61	Male	AoV 1 (100)	*L. jensenii* 1 (100)	Penicillin 1 (100) Aminopenicillin 1 (100) Aminoglycoside 1 (100)	Clinical cure 1 (100) Deaths overall 0 (0)
Chong et al., 1991 [36]	1	41	Male	MV 1 (100)	*L. casei* 1 (100)	Aminopenicillin 1 (100)	Clinical cure 1 (100) Deaths overall 0 (0)

Griffiths et al., 1992 [37]	2	31, 45	Male	AoV 2 (100)	*L. acidophilus* 1 (33.3) *L. rhamnosus* 1 (33.3)	Penicillin 1 (50) Aminopenicillin 1 (50) Quinolone 1 (50) Aminoglycoside 2 (100) Surgical management 1 (50)	Clinical cure 2 (100) Deaths overall 0 (0)
Sloss et al., 1993 [38]	1	79	Male	NR 1 (100)	*L. rhamnosus* 1 (100)	Aminopenicillin 1 (100) Aminoglycoside 1 (100) Surgical management 1 (100)	Clinical cure 1 (100) Deaths overall 0 (0)
Olearchyk et al., 1993 [39]	1	69	Male	AoV 1 (100) MV 1 (100)	*L. acidophilus* 1 (100) *L. acidophilus* 1 (100)	Penicillin 1 (100) Aminoglycoside 1 (100) Surgical management 1 (100)	Clinical cure 1 (100) Deaths overall 0 (0)
Puleo et al., 1994 [40]	1	21	Female	PV 1 (100)	*L. jensenii* 1 (100)	Penicillin 1 (100) Aminoglycoside 1 (100)	Clinical cure 1 (100) Deaths overall 0 (0)
Bessis et al., 1995 [41]	1	28	Female	MV 1 (100)	*L. acidophilus* 1 (100)	Penicillin 1 (100) Aminopenicillin 1 (100) Rifampicin 1 (100) Vancomycin 1 (100) Surgical management 1 (100)	Clinical cure 1 (100) Deaths overall 0 (0)
Jones et al., 1995 [42]	1	NR	NR	NR 1 (100)	*L. rhamnosus* 1 (100)	Aminopenicillin 1 (100) Macrolide 1 (100) Aminoglycoside 1 (100)	Clinical cure 0 (0) Deaths overall 1 (100) Deaths due to infection 1 (100)
Amrikachi et al., 1997 [43]	1	43	Male	MV 1 (100)	*L. acidophilus* 1 (100)	Aminopenicillin 1 (100) Cephalosporin 1 (100) Aminoglycoside 1 (100)	Clinical cure 0 (0) Deaths overall 1 (100) Deaths due to infection 1 (100)
Vaghijimal et al., 1997 [44]	1	80	Female	AoV 1 (100) MV 1 (100)	*L. jensenii* 1 (100)	Penicillin 1 (100) Aminoglycoside 1 (100)	Clinical cure 1 (100) Deaths overall 0 (0)

Mitchell et al., 1999 [45]	1	72	Male	MV 1 (100)	*L. curvatus* 1 (100)	Penicillin 1 (100) Aminoglycoside 1 (100)	Clinical cure 1 (100) Deaths overall 0 (0)
Mackay et al., 1999 [46]	1	67	Male	MV 1 (100)	*L. rhamnosus* 1 (100)	Penicillin 1 (100) Aminoglycoside 1 (100)	Clinical cure 1 (100) Deaths overall 0 (0)
Avlami et al., 2001 [47]	1	65	Male	AoV 1 (100)	*L. rhamnosus* 1 (100)	Penicillin 1 (100) Cephalosporin 1 (100) Quinolone 1 (100) Clindamycin 1 (100) Aminoglycoside 1 (100)	Clinical cure 1 (100) Deaths overall 0 (0)
Presterl et al., 2001 [48]	1	23	Male	AoV 1 (100)	*L. casei* 1 (100)	Penicillin 1 (100) Aminopenicillin 1 (100) Surgical management 1 (100)	Clinical cure 1 (100) Deaths overall 0 (0)
Schoevaerdts et al., 2002 [49]	1	82	Male	AoV 1 (100)	*Lactobacillus* spp. 1 (100)	Penicillin 1 (100) Clindamycin 1 (100) Clindamycin 1 (100) Aminoglycoside 1 (100)	Clinical cure 1 (100) Deaths overall 0 (0)
Wallet et al., 2002 [50]	1	73	Male	MV 1 (100)	*L. rhamnosus* 1 (100)	Aminopenicillin 1 (100) Vancomycin 1 (100) Rifampicin 1 (100) Aminoglycoside 1 (100) Surgical management 1 (100)	Clinical cure 1 (100) Deaths overall 0 (0)
Beldner et al., 2002 [51]	1	31	Female	MV 1 (100)	*Lactobacillus* spp. 1 (100)	Penicillin 1 (100) Aminoglycoside 1 (100) Surgical management 1 (100)	Clinical cure 1 (100) Deaths overall 0 (0)
Soleman et al., 2003 [52]	1	75	Female	AoV 1 (100)	*L. paracasei* 1 (100)	Penicillin 1 (100) Aminoglycoside 1 (100)	Clinical cure 1 (100) Deaths overall 0 (0)
Ze-Ze et al., 2004 [53]	1	53	Male	AoV 1 (100)	*L. acidophilus* 1 (100)	Antipseudomonal penicillin 1 (100) Tetracycline 1 (100) Aminoglycoside 1 (100) Surgical management 1 (100)	Clinical cure 1 (100) Deaths overall 0 (0)
Khan et al., 2005 [54]	1	16	Female	MV 1 (100)	*L. jensenii* 1 (100)	Aminopenicillin 1 (100)	Clinical cure 1 (100)

						Antipseudomonal penicillin 1 (100) Aminoglycoside 1 (100)	Deaths overall 0 (0)
Makaryus et al., 2005 [55]	1	63	Female	MV 1 (100)	*L. acidophilus* 1 (100)	Aminopenicillin 1 (100) Cephalosporin 1 (100) Carbapenem 1 (100) Vancomycin 1 (100) Macrolide 1 (100)	Clinical cure 1 (100) Deaths overall 0 (0)
Salvana et al., 2006 [56]	1	62	Female	NR 1 (100)	*L. acidophilus* 1 (100)	Penicillin 1 (100) Aminoglycoside 1 (100)	Clinical cure 1 (100) Deaths overall 0 (0)
See et al., 2006 [57]	1	42	Male	MV 1 (100)	*L. casei* 1 (100)	Aminopenicillin 1 (100) Aminoglycoside 1 (100) Surgical management 1 (100)	Clinical cure 1 (100) Deaths overall 0 (0)
Khasnis et al., 2006 [58]	1	42	Male	AoV 1 (100)	*L. casei* 1 (100)	Penicillin 1 (100) Vancomycin 1 (100) Quinolone 1 (100) Aminoglycoside 1 (100) Surgical management 1 (100)	Clinical cure 1 (100) Deaths overall 0 (0)
Yagi et al., 2008 [59]	1	42	Male	AoV 1 (100)	*Lactobacillus* spp. 1 (100)	Penicillin 1 (100) Aminoglycoside 1 (100) Surgical management 1 (100)	Clinical cure 1 (100) Deaths overall 0 (0)
Wolz et al., 2008 [60]	1	69	Male	NR 1 (100)	*L. rhamnosus* 1 (100)	NR 1 (100)	Clinical cure 0 (0) Deaths overall 1 (100) Deaths due to infection 1 (100)
Fradiani et al., 2010 [61]	1	47	Female	MV 1 (100)	*L. jensenii* 1 (100)	Penicillin 1 (100) Aminoglycoside 1 (100)	Clinical cure 1 (100) Deaths overall 0 (0)
Arshad et al., 2010 [62]	1	64	Male	AoV 1 (100)	*L. acidophilus* 1 (100)	Penicillin 1 (100) Aminoglycoside 1 (100)	Clinical cure 0 (0) Deaths overall 1 (100)

							Deaths due to infection 1 (100)
Nishijima et al., 2012 [63]	1	28	Female	MV 1 (100)	*Lactobacillus* spp. 1 (100)	Penicillin 1 (100)<br>Clindamycin 1 (100)	Clinical cure 1 (100)<br>Deaths overall 0 (0)
Suarez-Garcıa et al., 2012 [64]	1	27	Female	AoV 1 (100)	*L. jensenii* 1 (100)	Penicillin 1 (100)<br>Cephalosporin 1 (100)<br>Aminoglycoside 1 (100)<br>Surgical management 1 (100)	Clinical cure 1 (100)<br>Deaths overall 0 (0)
Walker et al., 2013 [65]	1	71	Male	AoV 1 (100)	*L. casei* 1 (100)	Penicillin 1 (100)<br>Vancomycin 1 (100)<br>Aminoglycoside 1 (100)<br>Surgical management 1 (100)	Clinical cure 1 (100)<br>Deaths overall 0 (0)
Franko et al., 2013 [66]	1	77	Male	MV 1 (100)	*L. paracasei* 1 (100)	Aminopenicillin 1 (100)<br>Aminoglycoside 1 (100)<br>Surgical management 1 (100)	Clinical cure 1 (100)<br>Deaths overall 0 (0)
Botros et al., 2014 [67]	1	17	Female	PV 1 (100)	*Lactobacillus* spp. 1 (100)	Penicillin 1 (100)<br>Cephalosporin 1 (100)<br>Clindamycin 1 (100)<br>Aminoglycoside 1 (100)	Clinical cure 1 (100)<br>Deaths overall 0 (0)
Marciniak et al., 2014 [68]	1	31	Female	AoV 1 (100)	*L. jensenii* 1 (100)	Teicoplanin 1 (100)<br>Rifampicin 1 (100)<br>Surgical management 1 (100)	Clinical cure 1 (100)<br>Deaths overall 0 (0)
Patnaik et al., 2015 [69]	1	56	Male	AoV 1 (100)	*L. jensenii* 1 (100)	Penicillin 1 (100)<br>Aminopenicillin 1 (100)<br>Antipseudomonal penicillin 1 (100)<br>Vancomycin 1 (100)<br>Surgical management 1 (100)	Clinical cure 1 (100)<br>Deaths overall 0 (0)
Gupta et al., 2015 [70]	1	65	Male	MV 1 (100)	*Lactobacillus* spp. 1 (100)	NR 1 (100)<br>Surgical management 1 (100)	Clinical cure 1 (100)<br>Deaths overall 0 (0)
Felekos et al., 2016 [71]	1	74	Male	MV 1 (100)	*L. rhamnosus* 1 (100)	Penicillin 1 (100)<br>Cephalosporin 1 (100)	Clinical cure 1 (100)

						Aminoglycoside 1 (100)<br>Surgical management 1 (100)	Deaths overall 0 (0)
Kato et al., 2016 [72]	1	78	Male	AoV 1 (100)	*L. paracasei* 1 (100)	Clindamycin 1 (100)<br>Surgical management 1 (100)	Clinical cure 1 (100)<br>Deaths overall 0 (0)
Encarnacion et al., 2016 [73]	1	48	Male	AoV 1 (100)	*L. acidophilus* 1 (100)	Penicillin 1 (100)<br>Antipseudomonal penicillin 1 (100)<br>Vancomycin 1 (100)<br>Daptomycin 1 (100)<br>Surgical management 1 (100)	Clinical cure 1 (100)<br>Deaths overall 0 (0)
Passera et al., 2016 [74]	1	2	NR	NR 1 (100)	*L. rhamnosus* 1 (100)	Cephalosporin 1 (100)<br>Vancomycin 1 (100)<br>Quinolone 1 (100)	Clinical cure 0 (0)<br>Deaths overall 1 (100)<br>Deaths due to infection 0 (0)
Stroupe et al., 2017 [75]	1	51	Male	MV 1 (100)	*L. zeae* 1 (100)	Penicillin 1 (100)<br>Antipseudomonal penicillin 1 (100)<br>Vancomycin 1 (100)<br>Aminoglycoside 1 (100)<br>Surgical management 1 (100)	Clinical cure 1 (100)<br>Deaths overall 0 (0)
Aaron et al., 2017 [76]	1	80	Male	AoV 1 (100)<br>MV 1 (100)	*L. rhamnosus* 1 (100)	Penicillin 1 (100)<br>Aminopenicillin 1 (100)<br>Vancomycin 1 (100)<br>Aminoglycoside 1 (100)<br>Surgical management 1 (100)	Clinical cure 1 (100)<br>Deaths overall 0 (0)
Groga-Bada et al., 2018 [77]	1	81	Female	MV 1 (100)	*Lactobacillus* spp. 1 (100)	Aminopenicillin 1 (100)<br>Antipseudomonal penicillin 1 (100)<br>Aminoglycoside 1 (100)<br>Surgical management 1 (100)	Clinical cure 1 (100)<br>Deaths overall 0 (0)
Naqvi et al., 2018 [78]	1	36	Female	AoV 1 (100)	*L. rhamnosus* 1 (100)	Penicillin 1 (100)<br>Vancomycin 1 (100)<br>Aminoglycoside 1 (100)	Clinical cure 0 (0)

						Surgical management 1 (100)	Deaths overall 1 (100)<br>Deaths due to infection 1 (100)
Lim et al., 2018 [79]	1	85	Male	MV 1 (100)	*L. garvieae* 1 (100)	Cephalosporin 1 (100)	Clinical cure 1 (100)<br>Deaths overall 0 (0)
Boumis et al., 2018 [80]	1	65	NR	AoV 1 (100)	*L. rhamnosus* 1 (100)	Aminopenicillin 1 (100)<br>Cephalosporin 1 (100)<br>Aminoglycoside 1 (100)	Clinical cure 1 (100)<br>Deaths overall 0 (0)
Ajam et al., 2019 [81]	1	75	Female	AoV 1 (100)	*L. paracasei* 1 (100)	Penicillin 1 (100)<br>Cephalosporin 1 (100)<br>Vancomycin 1 (100)<br>Aminoglycoside 1 (100)	Clinical cure 0 (0)<br>Deaths overall 1 (100)<br>Deaths due to infection 0 (0)
Osman et al., 2019 [82]	1	65	Male	AoV 1 (100)	*L. paracasei* 1 (100)	Penicillin 1 (100)<br>Cephalosporin 1 (100)<br>Vancomycin 1 (100)<br>Aminoglycoside 1 (100)	Clinical cure 1 (100)<br>Deaths overall 0 (0)
Agrawal et al., 2020 [83]	1	83	Male	AoV 1 (100)	*Lactobacillus* spp. 1 (100)	Aminopenicillin 1 (100)<br>Clindamycin 1 (100)<br>Aminoglycoside 1 (100)<br>Surgical management 1 (100)	Clinical cure 0 (0)<br>Deaths overall 1 (100)<br>Deaths due to infection 1 (100)
Antoun et al., 2020 [84]	1	40	Male	AoV 1 (100)	*L. rhamnosus* 1 (100)	Cephalosporin 1 (100)<br>Carbapenem 1 (100)<br>Vancomycin 1 (100)<br>Surgical management 1 (100)	Clinical cure 1 (100)<br>Deaths overall 0 (0)
Pasala et al., 2020 [85]	1	50	Male	AoV 1 (100)<br>MV 1 (100)	*L. rhamnosus* 1 (100)	Aminopenicillin 1 (100)<br>Cephalosporin 1 (100)<br>Aminoglycoside 1 (100)	Clinical cure 1 (100)<br>Deaths overall 0 (0)

						Surgical management 1 (100)	
Chukwurah et al., 2020 [86]	1	60	Male	AoV 1 (100)	*L. acidophilus* 1 (100)	Antipseudomonal penicillin 1 (100)<br>Co-trimoxazole 1 (100)<br>Surgical management 1 (100)	Clinical cure 1 (100)<br>Deaths overall 0 (0)
Campagne et al., 2020 [87]	1	57	Male	MV 1 (100)	*L. rhamnosus* 1 (100)	Aminopenicillin 1 (100)<br>Rifampicin 1 (100)<br>Aminoglycoside 1 (100)	Clinical cure 1 (100)<br>Deaths overall 0 (0)
Tavernese et al., 2020 [88]	1	48	Male	AoV 1 (100)	*L. plantarum* 1 (100)	Penicillin 1 (100)<br>Aminopenicillin 1 (100)<br>Aminoglycoside 1 (100)<br>Surgical management 1 (100)	Clinical cure 1 (100)<br>Deaths overall 0 (0)
Minto et al., 2020 [89]	1	57	Female	AoV 1 (100)	*L. jensenii* 1 (100)	Aminopenicillin 1 (100)<br>Macrolide 1 (100)<br>Surgical management 1 (100)	Clinical cure 1 (100)<br>Deaths overall 0 (0)
Ozer et al., 2020 [90]	1	42	Male	AoV 1 (100)	*L. acidophilus* 1 (100)	Penicillin 1 (100)<br>Cephalosporin 1 (100)<br>Vancomycin 1 (100)<br>Clindamycin 1 (100)<br>Surgical management 1 (100)	Clinical cure 1 (100)<br>Deaths overall 0 (0)
Campbell et al., 2020 [91]	1	45	Male	MV 1 (100)	*L. paracasei* 1 (100)	Penicillin 1 (100)<br>Cephalosporin 1 (100)<br>Vancomycin 1 (100)<br>Aminoglycoside 1 (100)<br>Surgical management 1 (100)	Clinical cure 1 (100)<br>Deaths overall 0 (0)
Bergas et al., 2021 [92]	2	81, 83	1 female<br>1 male	MV 1 (50)	*L. rhamnosus* 2 (100)	Penicillin 1 (50)<br>Aminopenicillin 1 (50)<br>Aminoglycoside 1 (50)	Clinical cure 2 (100)<br>Deaths overall 0 (0)
Pischel et al., 2021 [93]	1	67	Male	MV 1 (100)	*Lactobacillus* spp. 1 (100)	Penicillin 1 (100)<br>Aminopenicillin 1 (100)<br>Surgical management 1 (100)	Clinical cure 1 (100)<br>Deaths overall 0 (0)

Udongwo et al., 2022 [94]	1	77	Male	Left pulmonary vein 1 (100)	*Lactobacillus* spp. 1 (100)	NR 1 (100)<br>Surgical management 1 (100)	Clinical cure 0 (0)<br>Deaths overall 1 (100)<br>Deaths due to infection 1 (100)
Grazioli-Gauthier et al., 2022 [20]	1	40	Male	MV 1 (100)	*L. jensenii* 1 (100)	Aminopenicillin 1 (100)<br>Aminoglycoside 1 (100)<br>Surgical management 1 (100)	Clinical cure 1 (100)<br>Deaths overall 0 (0)
Bapna et al., 2023 [95]	1	22	Female	MV 1 (100)	*L. jensenii* 1 (100)	Carbapenem 1 (100)<br>Vancomycin 1 (100)<br>Surgical management 1 (100)	Clinical cure 1 (100)<br>Deaths overall 0 (0)
Rahman et al., 2023 [96]	1	71	Female	AoV 1 (100)<br>MV 1 (100)	*L. casei* 1 (100)	Aminopenicillin 1 (100)<br>Daptomycin 1 (100)<br>Surgical management 1 (100)	Clinical cure 1 (100)<br>Deaths overall 0 (0)
DeMarco et al., 2023 [97]	1	61	Female	TrV 1 (100)<br>CIED 1 (100)	*L. casei* 1 (100)<br>*L. paracasei* 1 (100)<br>*L. zeae* 1 (100)	Aminopenicillin 1 (100)<br>Carbapenem 1 (100)<br>Quinolone 1 (100)<br>Aminoglycoside 1 (100)<br>Surgical management 1 (100)	Clinical cure 1 (100)<br>Deaths overall 0 (0)

[a] Defined as the clinical resolution of the infection as a result of treatment. AoV: aortic valve; CIED: cardiac implantable electronic device; MV: mitral valve; PV: pulmonary valve TrV: tricuspid valve.

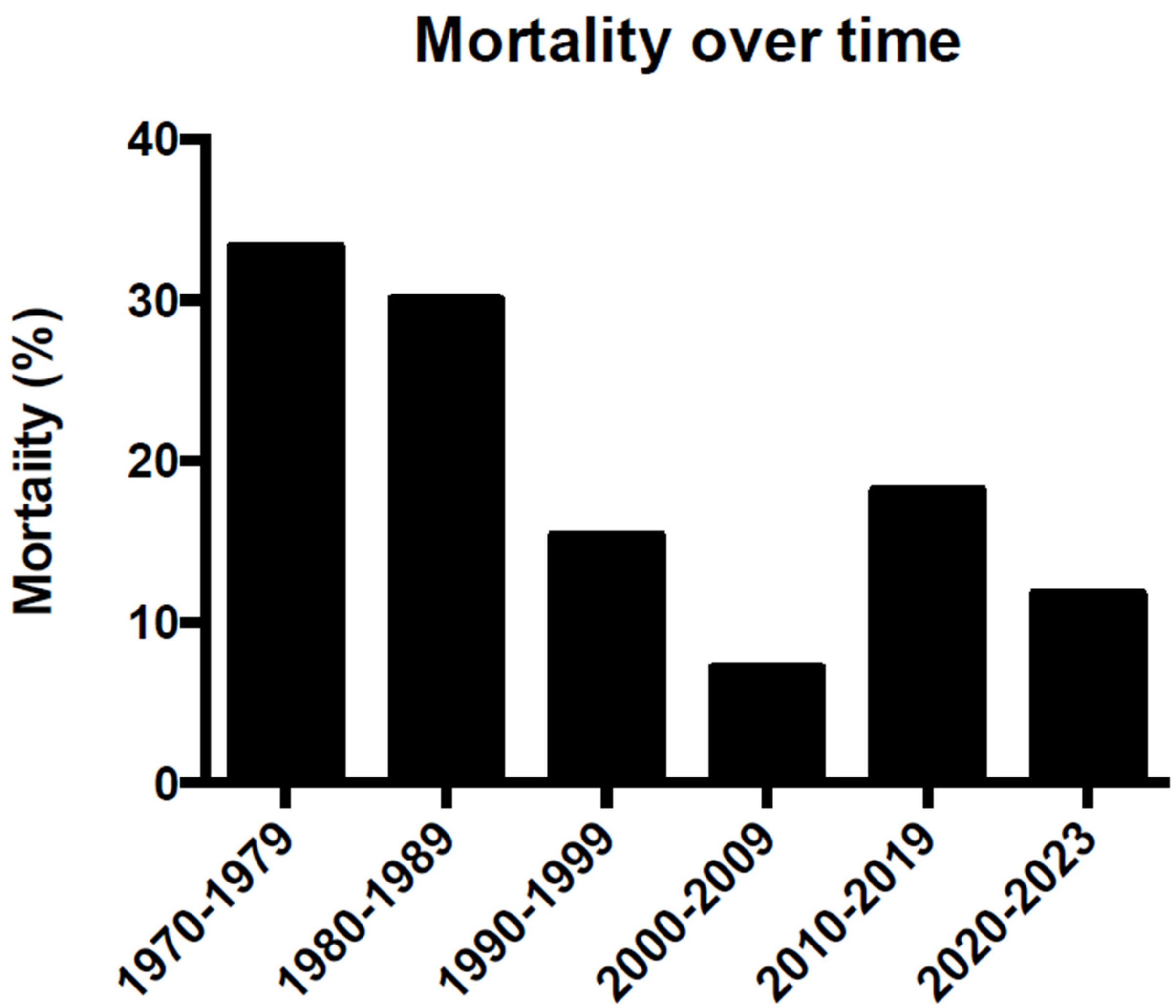

Figure S1. Overall mortality of patients with infective endocarditis by *Lactobacillus* species over time.
